# Impact of high pressure impregnation and air drying on the quality of *Dosidicus gigas* slices

**DOI:** 10.1038/s41598-025-87647-8

**Published:** 2025-01-30

**Authors:** Liliana Zura-Bravo, Roberto Lemus-Mondaca, Jaime Ortiz, Marcos Flores, Gipsy Tabilo-Munizaga, Mario Pérez-Won, Klaudia Masztalerz

**Affiliations:** 1 Laboratorio de Ciencias de los Alimentos, Facultad de Medicina y Ciencias de la Salud, Universidad Central de Chile, Santiago, CP 8330546 Chile; 2 Departamento de Ciencia de Alimentos y Tecnología Química, Facultad de Ciencias Químicas y Farmacéuticas, Universidad de Chile, St. Dr. Carlos Lorca 964, Independencia, Santiago, RM Chile; 3 Departamento de Horticultura, Facultad de Ciencias Agrarias, Universidad de Talca, Av. Lircay s/n, Talca, 3460000 Chile; 4 Departamento de Ingeniería en Alimentos, Facultad de Ciencias de la Salud y Alimentos, Universidad del Bío-Bío, Av. Andrés Bello s/n, Box 447, Chillán, Chile; 5 Institute of Agricultural Engineering, Wroclaw University of Environmental and Life Sciences, Chełmońskiego 37/41, Wroclaw, 51-630 Poland

**Keywords:** Humboldt squid, High-pressure impregnation, Moisture diffusivity, Color, Texture, Microstructure, Ocean sciences, Engineering

## Abstract

Humboldt squid (*Dosidicus gigas*) is the most abundant cephalopod in the fishing industry, and its high nutritional and organoleptic properties make it a go-to food product for consumers. Therefore, developing new processing techniques seems imperative to minimize quality deterioration and provide products with appropriate characteristics. The study aimed to determine the effect of high-pressure impregnation (HPI) pretreatment on hot air-drying kinetics and the quality of Humboldt squid slices. Various pressures, times, and concentrations of osmotic solution during HPI were evaluated, followed by drying at 40 and 60 °C. The HPI pretreatment reduced the drying time by around 26% when dried at 40 °C, and only 18% when dried at⋅ 60 °C compared with unpretreated samples. The Weibull, Page, and Logarithmic models were considered for experimental drying curve modeling. Diffusion coefficient values varied from 3.82 to 6.59 × 10^−9^ m^2^/s for all drying conditions. Moreover, the color, texture, and water-holding capacity were determined. Rehydration capacity values increased due to less damage to cellular tissue than the control (HPI-untreated dried samples). Also, scanning electron microscope (SEM) images showed a compacted structure of HPI-dried squid samples. Overall, HPI proved to be a beneficial pretreatment as it reduced drying time and improved the quality characteristics of Humboldt squid.

## Introduction

The *Dosidicus gigas*, commonly known as the Humboldt squid or jumbo squid in the USA, jibia in Chile, and pota in Peru, is the largest squid species in the Pacific Ocean^1^. Its substantial size and abundance have positioned it as a highly sought-after cephalopod within the fishing industry. This squid has a broad latitudinal range, extending over 10,000 km and potentially reaching Alaska and 45°S in the southeastern Pacific Ocean, from the sea surface to depths of 1200 m^2–4^. Therefore, this species thrives in broad habitat conditions, which is essential for studying its abundance and the circumstances under which the fishery industry can capture it. Recent years have seen a surge in the consumption of Humboldt squid, driven by its notable nutritional benefits, such as low-fat content and high-quality proteins, along with its appealing organoleptic qualities—characterized by a mild flavor and white color^5^. Consequently, the Humboldt squid is a prime candidate for developing various innovative, value-added products, boasting all the requisite characteristics to contribute significantly to this endeavor^6^.

Innovations in processing methods within the fishing industry have emerged to mitigate various physical, chemical, and biological reactions that lead to undesirable rapid deterioration^7^. While the osmotic dehydration technique has been utilized as a pretreatment to preserve food quality, the reduction in water activity following this treatment is often insufficient to prevent microbial growth. Numerous studies have explored the application of vacuum impregnation techniques in preserving various vegetable food materials, employing vacuum pressures ranging from 5 to 30 kPa and various impregnating substances, including sucrose, calcium lactate, citric acid, and ascorbic acid, among others^8–11^. This technique aims to enhance food’s texture, color, nutrient retention, and shelf-life. It has been employed as a pre-treatment before other processing methods, such as freezing or drying fruits and vegetables^12^. Notably, vacuum impregnation has also been successfully applied in the treatment of fish products, including abalone^13^, tilapia^14^, and seabream fillets^15^, among others^16^.

A combination of high hydrostatic pressure (HHP) (200 MPa − 500 MPa) with osmotic dehydration, referred to as high-pressure impregnation (HPI), has shown promise in dehydrating cells while increasing soluble solids until equilibrium is reached, thereby minimizing net transport phenomena^17^. This innovative technique has been applied to food materials of both animal and vegetable origin, leading to significant advancements^15,18–21^. Furthermore, the use of HPI pretreatment has been shown to enhance drying rates and improve the overall quality of dehydrated products, particularly when considering fishery products^17,22^. Cepero-Betancourt et al^23^. integrated HPI treatment with drying processes, significantly reducing drying time. The effect of osmotic dehydration temperature (75, 85, and 95 °C) on moisture loss and salt intake kinetics of jumbo squid with 6% NaCl (w/v) was evaluated by Uribe et al^22^. The study did not evaluate the quality properties of either drying condition. The same authors also performed prior research on HPI in squid; however, they only evaluated and predicted moisture and salt transfer kinetics^18^, i.e., the study did not consider drying nor the subsequent assessment of quality attributes.

Many physicochemical reactions happen during drying, causing deterioration of the product quality^24^. Thus, quality features (moisture content, water activity, texture profile, surface color, rehydration capacity, water holding capacity, and microstructure) should be measured and optimized to achieve a high-added-value marine product^25^. Based on the preceding, it is intended to strengthen the best processing technique and apply it to fishery products to improve quality features. Therefore, the influence of different high-pressure impregnation (HPI) conditions before the convective drying process on drying kinetics and physicochemical quality parameters of Humboldt squid (*Dosidicus gigas*) slices was evaluated in the current study.

## Materials and methods

### Preparation of the material

Around 15 kg of jumbo squid flesh (30–40 cm and 2–3 kg of each jumbo squid) were purchased from the fisheries market at Coquimbo City, Chile (29°57’19.9"S 71°20’08.7"W) to be processed in the current study. The external skin was removed from squid fillets, and the fillets were cut into slices of 5.0 ± 0.1 by 5.0 ± 0.1 by 1.0 ± 0.1 cm (length × width × thickness). The samples were meticulously cut with a fruit/vegetable chopper (stainless steel knives) to ensure precise control over the thickness approach. Ten squid slices were obtained from each squid. Then, the samples were refrigerated at 4.0 ± 0.2 °C in polyethylene pouches for no more than 48 h until further analysis and processing.

The proximal analysis was carried out by different AOAC (1990) methodologies (moisture content: AOAC 934.06; crude protein content: AOAC 960.52; lipid content: AOAC 960.39; crude ash content: AOAC 923.03)^26^. The Mohr method determined the salt content (g NaCl/100 g sample). A water activity meter (Novasina TH-500, Lachen, Switzerland) was used to determine the water activity (*a*_*w*_) of the samples. The experiments were performed in triplicate.

### HPI pretreatment

The osmotic solutions were prepared using commercial salt and distilled water (NaCl concentrations of 10 and 15% w/v at room temperature: ±20 °C) in polyethylene (PE) bags. Then, the squid fillets were individually weighed, submerged at the same time in PE bags with a 1/8 (w/w) squid-to-brine ratio solution, and thermo-sealed before high-pressure processing. A pilot high-pressure unit with a 2 L cylindrical loading container (Avure Technologies Inc., Kent, WA, USA) was used for high-pressure treatment. The pressurizing medium (water), working at 17 MPa/s ramp rate and 5 s decompression time at room temperature (± 25 °C) was used to develop the following conditions: two pressure levels, 350 and 550 MPa, and two pressure times, 5 and 10 min. After being pressurized, the squid samples were removed from the solution, and tissue paper was used to eliminate excess brine from the surface. The HPI operating conditions were based on the previous research of Lemus-Mondaca et al^18^., in which they evaluated the effect of different pressures (100–400 MPa) at 15% NaCl on the mass transfer kinetics of moisture and solids.

### Drying process

**Fig. 1:**
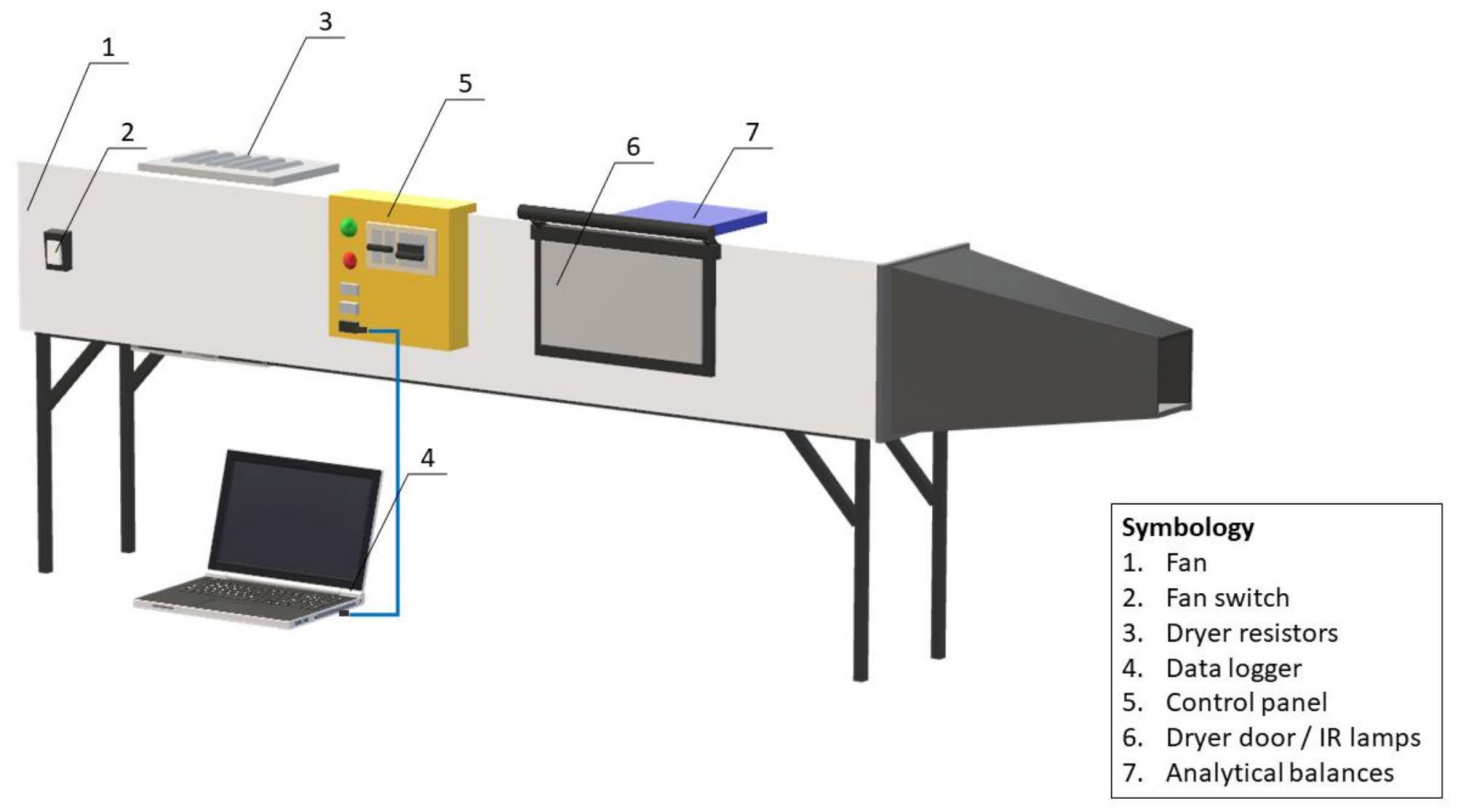
Schematic diagram of drying equipment, Autodesk AutoCAD 2020 (version Education Community, University of Chile).

The experimental setup involved drying control samples at 40 and 60 °C without osmotic pretreatment, which were then compared to (HPI)-pretreated samples during runs 1–16. Both control and HPI-pretreated samples were promptly placed into a hot-air convective dryer custom-designed by the Food Science Department at Universidad de Chile (Fig. 1). This dryer featured a control unit to regulate the air inlet velocity and temperature, heated via electrical resistances. Two temperatures, 40 and 60 °C, were utilized, maintaining a constant airflow of 2.0 ± 0.2 m/s, with each experiment conducted in triplicate. Samples (102.4 ± 1.2 g) were arranged in a thin layer in a stainless-steel basket. The weight loss was measured on an analytical balance, ± 0.01 g (SP402, Ohaus, China), at defined intervals and connected by a system interface (RS232, Ohaus, China) to a personal computer, recording and storing the weight decrease data. The experiments were finished at reaching equilibrium conditions (i.e., constant weight). All dried samples, including the control, were vacuum-packed into polyethylene bags for subsequent quality analysis.

### Diffusion coefficient and modeling

The moisture ratio (MR) relates the sample moisture content at a given time *X*_*t*_ with the initial moisture content *X*_*0*_ of the sample and the equilibrium moisture content *X*_*e*_ (Eq. 1). Then, a diffusion coefficient (*D*_*we*_) was obtained based on the integrated equation of Fick’s second law (Eq. 2), and Eq. 3representing the first term in the development of the series^27^. Fick’s second diffusion law predicts the drying process during the falling rate period when water is transported to the material’s surface by diffusion^28–32^.

1 $$MR = \frac{{X_{t} - X_{e} }}{{X_{o} - X_{e} }}$$

2 $$MR = \frac{8}{{\pi^{2} }}\sum\limits_{i = 0}^{\infty } {\frac{1}{{\left( {2\,i + 1} \right)^{2} }}{\text{exp}}} \;\left[ {\frac{{ - \left( {2\,i + 1} \right)^{2} D_{we} \,\pi^{2} \,t}}{{4L^{2} }}} \right]$$

3 $$MR = \frac{8}{{\pi^{2} }}{\text{exp}}\left[ {\frac{{ - D_{we} \,\pi^{2} \,t}}{{4L^{2} }}} \right]$$

Various mathematical models can be used to predict the drying curve of experimental data obtained during drying of food and agricultural products^22,33,34^. Thus, the Page, Weilbull, and Logarithmic equations (Eqs. 4–6, accordingly) were applied in this study.

4 $$MR = \exp \,\left( { - kt^{n} } \right)$$

5 $$MR = \exp \,\left( {\,\left( { - {t \mathord{\left/ {\vphantom {t \beta }} \right. \kern-0pt} \beta }} \right)^{\alpha } } \right)$$

6 $$MR = a \cdot \exp \,\left( { - kt} \right) + c$$

### Surface color

Evaluation of color changes in squid samples surface was performed using a HunterLab colorimeter device (MiniScan XE Plus, Reston, VA, USA), expressed in a CIE*Lab* three-dimensional space, along with standard illuminate D_65_ and observer 10°. The L* axis is the lightness and ranges from 0 (black) to 100 (white). The other two coordinate axes are a* and b*, representing variation between redness-greenness and yellowness-blueness, respectively. Total color difference (*ΔE*) and whiteness index (WI) were calculated using Eqs. (7) and (8), respectively, where *L*_*o*_, *a*_*o*_, and *b*_*o*_ are the control values. The experiments were done in triplicate.

7 $$\Delta E = \sqrt {\left( {L^* - L_{o} } \right)^{2} + \left( {a^* - a_{o} } \right)^{2} + \left( {b^* - b_{o} } \right)^{2} }$$

8 $$WI=100-\sqrt{{\left(100-{L}^{*}\right)}^{2}{+\left({a}^{*}\right)}^{2}{\left({b}^{*}\right)}^{2}}$$

### Texture profile analysis

The squid fillets were manually cut into 20.0 ± 1.5 cm^3^cubes. The samples were rehydrated in distilled water for 15 min at 20 °C. The instrumental texture profile analysis (TPA) of squid samples was performed with a TA-TX PLUS texture analyzer (Texture Tech., Scarsdale, NY, USA) along with Texture Expert v.2.63 software (Stable Micro Systems Ltd.). The analysis conditions were: (i) double compression of samples to 75% of their original thickness, (ii) constant compression speed of 1 mm/s, and (iii) applicable stainless-steel plate probe (35 mm in diameter). The texture parameters analyzed and recorded were hardness (gf), cohesiveness (-), springiness (mm), and chewiness (mm)^35^. Ten replicated instrumental measurements were performed for each analysis.

### Rehydration indexes

To determine the rehydration capacity (RC), the dried samples were placed in distilled water (solid-to-liquid ratio of 1:50) at 40 °C for 6 h. Then, the samples were removed, drained, and weighed. RC was obtained according to Eq. (9) and expressed in grams of absorbed water per gram of dry matter. Then, the water holding capacity (WHC) was determined according to Eq. (10) by centrifuging the rehydrated samples (the squid fillets manually cut into 20.0 ± 1.5 cm^3^ cubes) at 3500xg for 15 min at 20 °C in tubes specially adapted (*n* = 3) and expressed as g of retained water per 100 gram of water. These tubes had a steel-stainless mesh tightened centrally, which allowed the sample water to be drained.

9 $$RC = \frac{{\left( {W_{reh} \times X_{reh} } \right) - \left( {W_{dried} \times X_{dried} } \right)}}{{W_{dried} \times \left( {1 - X_{dried} } \right)}}$$

10 $$WHC = \frac{{\left( {W_{reh} \times X_{reh} } \right) - W_{dl} }}{{\left( {W_{reh} \times X_{reh} } \right)}}$$

*W*_*reh*_ is rehydrated sample weight (g), *X*_*reh*_ is rehydrated sample moisture content (w.b.), *W*_*dried*_ is dried sample weight (g), *X*_*dried*_ is dried sample moisture content, and *W*_*dl*_ is drained liquid weight after centrifugation (g).

### Microstructure analysis

A cryo-stage CT1500C (Oxford Instruments, Witney, UK) connected to a JEOL-JSM-5410 electron microscope (JEOL, Tokyo, Japan) was utilized for examining the microstructure of both unpretreated dried and HPI-dried squid samples. All samples underwent a rehydration process for 6 h at 20 ± 0.2 °C to prepare them for subsequent microstructure analysis. Then, the rehydrated samples were cryo-fixed by immersion in slush nitrogen (−210 °C) and promptly transferred to the cryo-stage at 1 kPa for fracturing. Sublimation was conducted at −90 °C, with pressures ranging from 1.33 to 0.67 kPa, for 15 min, with the final point determined through direct microscopic observation (5 kV). Following this, the samples underwent gold-coating within the cryo-stage unit using an ionization current of 2 mA, with an applied vacuum (0.2 kPa) for 3 min, and were then examined using Scanning Electron Microscopy (SEM) under cold-stage conditions. The fractured surface was observed while the temperature was maintained at approximately − 150 °C. Observations were carried out at 10 kV, with a working distance of 15 mm, and micrographs were taken at 300× magnification to discern changes in cell structure.

### Statistical evaluation and experimental design

The determination coefficient (R^2^), Sum Squared Error (SSE, Eq. 11), and Chi-squared (*χ*^*2*^, Eq. 12) were applied to evaluate the experimental data obtained in the study and determine the best fit of the mathematical models^36^. Statistical analysis was performed using Statgraphics Plus^®^ software v.5.1, applying an analysis of variance (ANOVA) to estimate the least significant differences (LSD) (*p* < 0.05). A Multiple Range Test (MRT) was used to determine homogeneous groups for diffusion coefficients and kinetic parameters.

11 $$SSE = \frac{1}{N}\sum\limits_{i = 0}^{N} {\left( {MR_{{\text{e,i}}} - MR_{c,i} } \right)\,^{2} }$$

12 $$\chi^{2} = \frac{{\sum\nolimits_{i = 0}^{N} {\left( {MR_{{\text{e,i}}} - MR_{c,i} } \right)}^{2} }}{N - z}$$

where: *MR*_*e, i*_ is the experimental moisture content; *MR*_*c, i*_ is the calculated moisture content; *i* is the number of terms; *z* is a constant number, and *N* is the data.

A multi-factorial design (2^4^) was used to develop the experimental design presented in Table 1. In this case, the four factors (*k* = 4) to evaluate were high pressure (HP = 350 and 550 MPa), pressure time (PT = 5 and 10 min), osmotic solution (OS, NaCl = 10 and 15%), and drying temperature (DT = 40 and 60 °C), and each with two levels (*n* = 2).

**Table 1:** Matrix levels of the operative variables.

Runs	DT	HP	PT	OS	Combination
1	−1	−1	−1	−1	40 °C, 350 MPa, 5 min, 10%
2	−1	−1	−1	+ 1	40 °C, 350 MPa, 5 min, 15%
3	−1	−1	+ 1	−1	40 °C, 350 MPa, 10 min, 10%
4	−1	−1	+ 1	+ 1	40 °C, 350 MPa, 10 min, 15%
5	−1	+ 1	−1	−1	40 °C, 550 MPa, 5 min, 10%
6	−1	+ 1	−1	+ 1	40 °C, 550 MPa, 5 min, 15%
7	−1	+ 1	+ 1	−1	40 °C, 550 MPa, 10 min, 10%
8	−1	+ 1	+ 1	+ 1	40 °C, 550 MPa, 10 min, 15%
9	+ 1	−1	−1	−1	60 °C, 350 MPa, 5 min, 10%
10	+ 1	−1	−1	+ 1	60 °C, 350 MPa, 5 min, 15%
11	+ 1	−1	+ 1	−1	60 °C, 350 MPa, 10 min, 10%
12	+ 1	−1	+ 1	+ 1	60 °C, 350 MPa, 10 min, 15%
13	+ 1	+ 1	−1	−1	60 °C, 550 MPa, 5 min, 10%
14	+ 1	+ 1	−1	+ 1	60 °C, 550 MPa, 5 min, 15%
15	+ 1	+ 1	+ 1	−1	60 °C, 550 MPa, 10 min, 10%
16	+ 1	+ 1	+ 1	+ 1	60 °C, 550 MPa, 10 min, 15%

The decoded values shown for the technological variables are DT (drying temperature): 40 °C (−1) and 60 °C (+ 1); HP (high pressure): 350 MPa (−1) and 550 MPa (+ 1); PT (pressure time): 5 min (−1) and 10 min (+ 1); OS (osmotic solution): 10% NaCl (−1) and 15% NaCl (+ 1).

## Results and discussion

### Proximate composition, salt content and water activity of samples

Physicochemical analysis of fresh squid presented an initial moisture content of 88.31 ± 0.69 g/100 g sample; crude protein (*N*×6.25) of 10.42 ± 0.80 g/100 g sample; total lipids of 0.10 ± 0.01 g/100 g sample; ash of 1.06 ± 0.04 g/100 g sample and salt content of 1.92 ± 0.01 g NaCl/100 g sample as well as initial water activity of 0.942 ± 0.002, and pH of 5.980 ± 0.006. Abugoch et al^37^., Cortés-Ruiz et al^38^., and Uribe et al.^22^ reported similar results.

### Mass diffusion coefficients (*D*_*we*_)

**Fig. 2:**
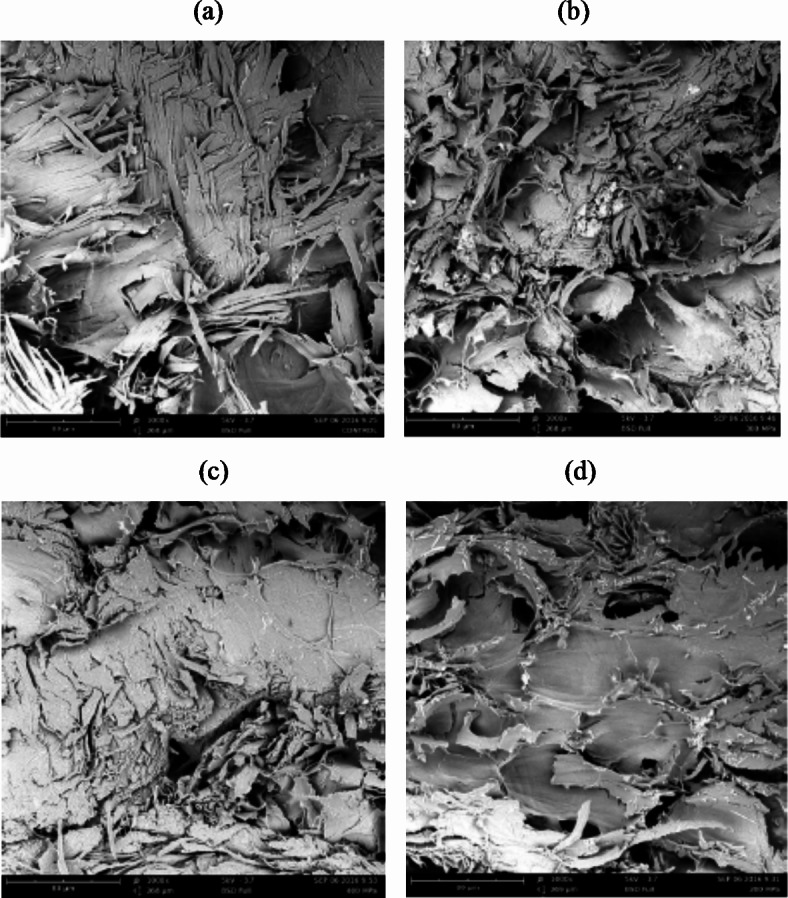
Cryo-SEM images 300× for the squid samples: (**a**) Control 40 °C, (**b**) Control 60 °C, (**c**) Run-9 (60 °C, 350 MPa, 5 min, 10%), and (**d**) Run-11 (60 °C, 350 MPa, 10 min, 10%).

Table 2 illustrates the mass diffusion coefficient values observed throughout the drying process for all HPI-pretreated dried squid samples, ranging from 3.82 to 6.59 × 10^−9^ m^2^/s (R^2^ ≥ 0.98). In contrast, the diffusion coefficients (*D*_*we*_) for unpretreated dried samples were measured at 1.76 ± 0.05 × 10^−9^ m^2^/s for 40 °C and 5.16 ± 0.72 × 10^−9^ m^2^/s for 60 °C. Notably, the *D*_*we*_ values at both temperatures were lower than those observed for HPI-pretreated dried samples, considering Runs 1–8 and 9–16, respectively. The influence of drying temperature on *D*_*we*_ was apparent, as an increase in this operational variable, from 40 °C to 60 °C, resulted in a corresponding rise in *D*_*we*_for both unpretreated and HPI-dried samples. Elevated temperatures were observed to facilitate faster water loss by inducing swelling and plasticization of cell membranes, thus enhancing mass transfer characteristics on the product surface^39^. Additionally, the application of high pressure led to modifications in the cell wall structure, rendering the cells more permeable and consequently bringing about significant changes in tissue architecture (Fig. 2). This, in turn, resulted in increased mass transfer rates during dehydration^17,40^. Also, from the same Table 2, the treated samples showed water activity values below 0.7, which inhibits microorganisms’ growth, with molds generally involved in spoilage at or above 0.7. The treated samples showed salt content ranging from 7.65 to 13.58%. In addition, salted squid is commonly eaten and has a salt content of about 25–30%, but salted squid for seasoning has a shallow salt content of less than 8%^41^.

**Table 2:** Effect of the processing conditions on equilibrium moisture (*X*_*e*_) and salt content (*X*_*s*_), water activity (*a*_*w*_), and mass diffusion coefficient (*D*_*we*_) of HPI-dried and control samples.

Runs	X_e_	X_s_	a_w_	D_we_
C40	0.346 ± 0.017 ^a^	2.23 ± 0.04 ^a^	0.652 ± 0.001 ^a^	1.76 ± 0.05 ^c^
R1	0.673 ± 0.018 ^c^	7.93 ± 0.06 ^b^	0.578 ± 0.005 ^c^	3.82 ± 0.37 ^a^
R2	0.830 ± 0.090 ^d^	11.13 ± 0.50 ^d^	0.596 ± 0.005 ^f^	4.00 ± 0.05 ^ab^
R3	0.620 ± 0.034 ^c^	7.74 ± 0.29 ^b^	0.554 ± 0.003 ^e^	4.02 ± 0.11 ^ab^
R4	0.553 ± 0.013 ^b^	13.58 ± 0.94 ^e^	0.546 ± 0.008 ^e^	4.09 ± 0.11 ^ab^
R5	0.617 ± 0.084 ^c^	7.65 ± 0.10 ^b^	0.568 ± 0.001 ^d^	3.99 ± 0.13 ^ab^
R6	0.751 ± 0.016 ^a^	10.04 ± 0.04 ^c^	0.587 ± 0.002 ^c^	4.07 ± 0.03 ^ab^
R7	0.657 ± 0.068 ^c^	7.85 ± 0.06 ^b^	0.582 ± 0.003 ^c^	4.15 ± 0.15 ^b^
R8	0.865 ± 0.032 ^d^	9.81 ± 0.13 ^c^	0.624 ± 0.001 ^b^	3.97 ± 0.13 ^ab^
C60	0.339 ± 0.009 ^A^	2.88 ± 0.12 ^A^	0.769 ± 0.002 ^D^	5.16 ± 0.72 ^D^
R9	0.577 ± 0.032 ^D^	7.83 ± 0.17 ^B^	0.704 ± 0.001 ^B^	6.11 ± 0.31 ^A^
R10	0.468 ± 0.007 ^C^	11.17 ± 0.08 ^E^	0.672 ± 0.001 ^A^	6.39 ± 0.18 ^B^
R11	0.484 ± 0.080 ^C^	8.38 ± 0.04 ^C^	0.720 ± 0.002 ^C^	6.38 ± 0.05 ^AC^
R12	0.885 ± 0.033 ^E^	11.39 ± 0.19 ^E^	0.716 ± 0.002 ^C^	6.62 ± 0.20 ^C^
R13	0.414 ± 0.014 ^B^	7.92 ± 0.13 ^B^	0.671 ± 0.004 ^A^	6.41 ± 0.25 ^AC^
R14	0.941 ± 0.024 ^F^	10.42 ± 0.08 ^D^	0.718 ± 0.002 ^C^	6.50 ± 0.08 ^AC^
R15	0.567 ± 0.022 ^D^	8.99 ± 0.56 ^C^	0.675 ± 0.003 ^A^	6.59 ± 0.57 ^C^
R16	1.096 ± 0.002 ^G^	11.81 ± 0.06 ^E^	0.703 ± 0.002 ^B^	6.40 ± 0.16 ^AC^

*X*_*e*_ (g water/g d.m.); *X*_*s*_ (g NaCl/100 g sample); *a*_*w*_ (dimensionless) and *D*_*we*_×10^−9^ (m²/s). Different lowercase letters (a, b, c) show significant differences during the effect of OS to drying at 40 °C under HP and PT constant conditions (Run:1–8). Different uppercase letters (A, B, C) show significant differences during the effect of OS to drying at 60 °C under HP and PT constant conditions (Run: 9–16). C40: Control 40 °C; C60: Control 60 °C; DT: drying temperature; HP: high pressure; PT: pressure time; OS: osmotic solution.

Several authors reported comparable results working with osmotic pre-treatments to a drying process in fish and squid products. Martins and da Silva Pena^36^ studied the osmotic dehydration under vacuum (15–25% NaCl, 7–101 kPa) and complementary drying of pirarucu (*Arapaima gigas*) fillets. They found effective diffusivity values ranging from 10.85 to 12.30 × 10^−9^ m^2^/s for drying temperatures 40 and 70 °C. In the study by Vega-Gálvez et al^42^., who also evaluated osmotic dehydration (6%NaCl, 85 °C) and convective drying (50–90 °C) for Jumbo squid fillets, the obtained *D*_*we*_ values varied from 0.78 to 3.2 × 10^−9^ m^2^/s. Conversely, Jain and Pathare^43^ reported *D*_*we*_ values of 1.11 and 8.708 × 10^−11^ m^2^/s for sun-dried prawn and chelwa fish, respectively.

The variations observed can be attributed to factors such as the diversity of seafood species, temperature variations, muscle orientation, fat content, and the presence or absence of skin^44^, as well as differences in pre-treatment conditions. As previously mentioned, all pretreated samples exhibited faster drying rates compared to control samples. Consequently, a multiple range test (MRT) was conducted on *D*_*we*_ for Runs 1–8 (samples dried at 40 °C) and 9–16 (samples dried at 60 °C). Analysis revealed that even though, variables such as high pressure (HP), pre-treatment (PT), and osmotic dehydration (OS) had a significant effect on *D*_*we*_ (*p* > 0.05) compared to control, the differences between the treatments were significant only in a few instances. It shows that more studies need to be done to better understand the effect of these variables on *D*_*we*_.

### Drying kinetics modeling

**Fig. 3:**
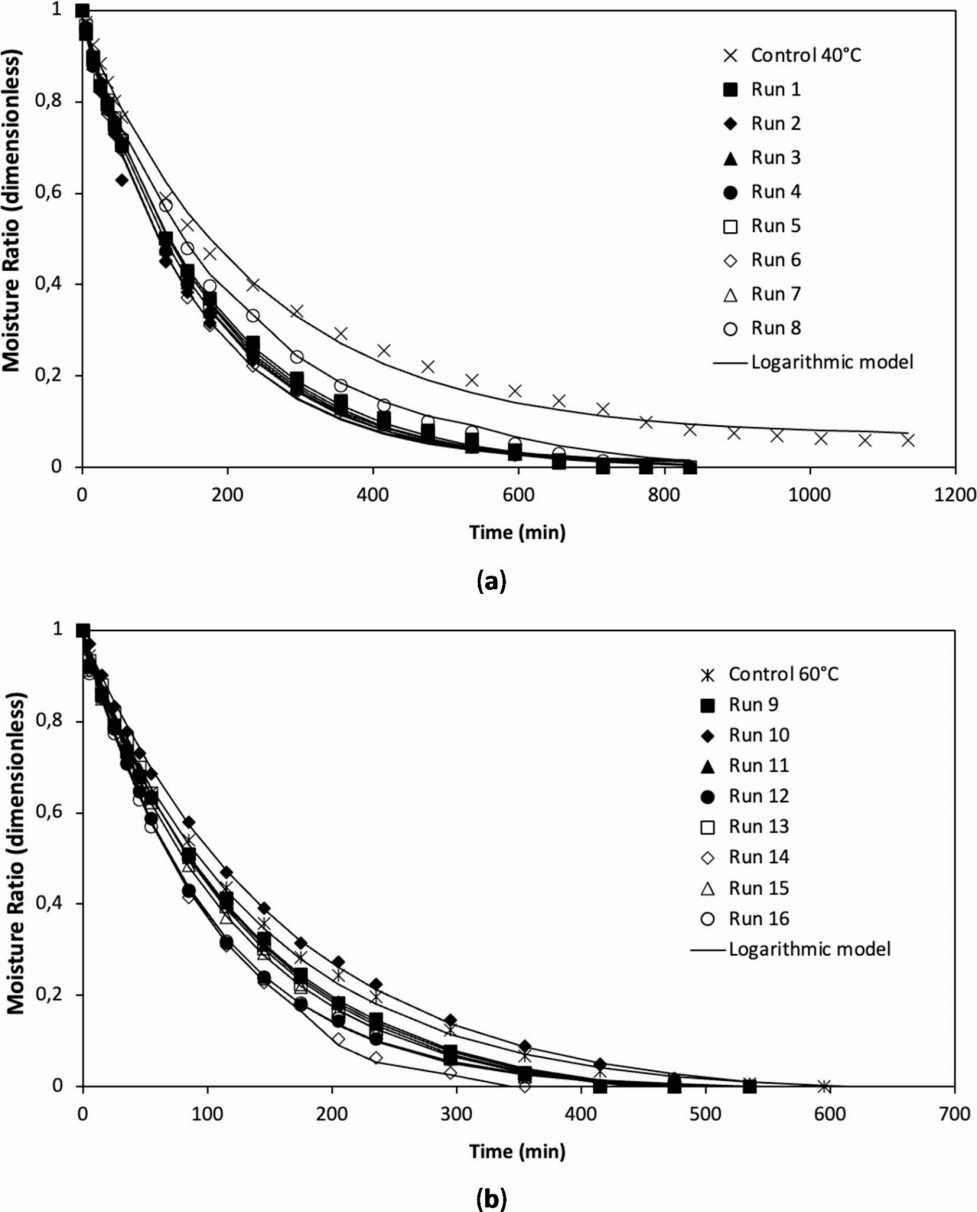
Drying kinetics and Logarithmic model values for (**a**) C40-R1-R8 and (**b**) C60-R9-R16 experimental runs (see Table 1).

The profiles of the experimental moisture ratio versus the drying time of squid samples for all drying treatments are presented in Fig. 3. It can be observed the curves exhibited an exponential behavior, which is typical for food drying curves^42^. The highest impact on the decrease of the moisture ratio was reported for drying temperature, followed by pressure time. Higher osmotic concentration and pressure affected the moisture ratio to a lower extent. In the case of 40 and 60 °C drying temperatures, lower moisture ratios were obtained for the pretreated samples compared to the control. Overall, pretreated samples required a shorter drying time than the control. It can be explained by the use of HPI pretreatment that improved the moisture diffusion rate in the samples (Table 2)^21,45^. Similar trends in terms of drying method and temperature were found in the studies by Duc and Toan^46^, in which they evaluated the effect of different drying methods at 40, 45, and 50 °C on the drying behavior of squid, including hot air drying (without osmotic pre-treatment).

Weibull, Page, and Logarithmic models were used to fit the experimental data of the drying process of squid slices (Fig. 3; Table 3). Modeling of control sample data (40 and 60 °C) revealed high values of the coefficient of determination for all mathematical models used in the study (R^2^ > 0.995). The ANOVA analysis didn’t show statistically significant differences (*p* > 0.05) for a (Logarithmic), n (Page), and α (Weibull) parameters. However, it was observed that for the k and c (Logarithmic), k and n (Page), and α and β (Weibull) parameters, a statistically significant difference (*p* < 0.05) was obtained. Like the diffusion coefficient, these parameters presented the same tendency regarding osmotic concentration. Thus, it may be assumed that these empirical parameters would be only directly proportional to the osmotic concentration.

**Table 3:** Kinetic and empirical parameters and their respective statistical tests for each mathematical model.

Run	Logarithmic			Statistical test			Weibull		Statistical test			Page		Statistical test		
	a	k	c	*R* ^2^	SSE	χ^2^	α	β	*R* ^2^	SSE	χ^2^	k	*n*	*R* ^2^	SSE	χ^2^
C40	0.974^a^	0.005	− 0.002^abc^	0.9988	0.0001	0.0001	0.896^a^	168.6^bc^	0.9694	0.0001	0.0001	0.010^a^	0.896^a^	0.9694	0.0001	0.0001
1	0.974^a^	0.005^b^	− 0.002^abc^	0.9988	0.0001	0.0001	0.896^a^	168.7^bc^	0.9694	0.0001	0.0001	0.010^a^	0.896^a^	0.9694	0.0001	0.0001
2	0.963^a^	0.006^d^	0.011^d^	0.9941	0.0003	0.0003	0.846^a^	179.2^c^	0.9322	0.0014	0.0016	0.014^a^	0.847^a^	0.9322	0.0007	0.0008
3	0.986^a^	0.005^b^	− 0.001^a^	0.9978	0.0008	0.0000	0.877^a^	176.7^c^	0.9351	0.0004	0.0005	0.010^a^	0.877^a^	0.9351	0.0006	0.0007
4	0.975^a^	0.006^bcd^	0.001^bcd^	0.9987	0.0001	0.0001	0.864^a^	174.6^c^	0.9382	0.0004	0.0005	0.012^a^	0.851^a^	0.9344	0.0004	0.0005
5	0.989^a^	0.005^b^	− 0.003^ab^	0.9988	0.0000	0.0000	0.904^a^	185.8^c^	0.9428	0.0005	0.0005	0.009^a^	0.904^a^	0.9428	0.0005	0.0005
6	0.970^a^	0.006^cd^	0.008^cd^	0.9983	0.0001	0.0001	0.847^a^	141.5^b^	0.9356	0.0005	0.0005	0.013^a^	0.847^a^	0.9356	0.0006	0.0007
7	0.980^a^	0.005^bc^	− 0.001^abc^	0.9945	0.0000	0.0001	0.818^a^	96.27^a^	0.9254	0.0100	0.0117	0.023^b^	0.818^a^	0.9254	0.0111	0.0124
8	0.975^a^	0.004^a^	− 0.013^a^	0.9975	0.0019	0.0022	0.824^a^	93.91^a^	0.9928	0.0127	0.0148	0.023^b^	0.824^a^	0.9928	0.0133	0.0148
C60	1.004^AB^	0.007^B^	− 0.034^A^	0.9985	0.0001	0.0001	0.898^BC^	116.2^E^	0.9463	0.0007	0.0008	0.014^A^	0.898^BC^	0.9463	0.0007	0.0008
9	1.004^AB^	0.007^B^	− 0.034^A^	0.9985	0.0001	0.0001	0.898^BC^	116.2^E^	0.9463	0.0007	0.0008	0.014^A^	0.898^BC^	0.9463	0.0007	0.0008
10	1.014^AB^	0.006^A^	− 0.026^A^	0.9992	0.0000	0.0001	0.840^AB^	61.21^B^	0.9899	0.2092	0.2391	0.031^B^	0.840^AB^	0.9899	0.0405	0.0459
11	1.014^AB^	0.007^B^	− 0.035^A^	0.9986	0.0000	0.0001	0.850^AB^	70.99^C^	0.9921	0.0149	0.0169	0.026^B^	0.850^AB^	0.9921	0.0149	0.0169
12	0.996^AB^	0.009^C^	− 0.007^B^	0.9988	0.0000	0.0000	0.864^AB^	63.35^BC^	0.9891	0.0112	0.0127	0.028^B^	0.864^AB^	0.9891	0.0112	0.0127
13	1.028^B^	0.007^B^	− 0.033^A^	0.9990	0.0000	0.0001	1.013^D^	109.2^E^	0.9714	0.0006	0.0007	0.009^A^	1.013^D^	0.9713	0.0009	0.0010
14	1.019^AB^	0.009^C^	− 0.028^A^	0.9984	0.0002	0.0003	0.961^CD^	94.59^D^	0.9727	0.0004	0.0005	0.012^A^	0.961^CD^	0.9727	0.0004	0.0004
15	1.008^AB^	0.007^B^	− 0.029^A^	0.9984	0.0001	0.0001	0.828^AB^	57.79^AB^	0.9878	0.0225	0.0239	0.035^B^	0.828^AB^	0.9878	0.0225	0.0239
16	0.982^A^	0.009^C^	− 0.004^B^	0.9988	0.0001	0.0001	0.791^A^	48.99^A^	0.9816	0.0226	0.0256	0.046^C^	0.791^A^	0.9816	0.0213	0.0240

C40: Control 40 °C; C60: Control 60 °C. Different lowercase letters (a, b, and c) show significant differences in the effect of OS on drying temperature 40 °C at a constant HP and PT (run:1–8). Different uppercase letters (A, B, and C) show significant differences in the effect of OS on drying temperature 60°Cat HP and PT constant (run: 9–16).

Statistical evaluation of mathematical models (Logarithmic, Page, and Weibull) showed low values of SSE and *χ*^*2*^ (Table 3) when applied to all experimental runs 1–16. Also, the same models showed low values of SSE (0.020–0.113) and *χ*^*2*^ (0.022–0.126) for control (40 and 60 °C). Among the considered models, the Logarithmic model showed the highest fit to the experimental data obtained in the study (0.9945 < R^2^ < 0.999; 0.0000 < SSE < 0.0019, and 0.0000 < *χ*^*2*^ < 0.0022). Therefore, Fig. 3compares experimental and modeled drying curves using the Logarithmic model. It is consistent with the study by Pérez-Won et al^20^., who worked with abalone osmotically dehydrated under high pressure followed by the drying process. Martins and da Silva Pena^36^ found that the Midilli-Kucuk and Page models were the best-predicting models for the drying kinetics of pirarucu (*Arapaima gigas*) fillets. Low values of SSE and *χ*^*2*^ imply an excellent fit to the experimental data and suggest its usefulness for predicting and modeling the drying kinetics during the hot air drying of squid samples under different HPI pre-treatments.

### Surface color changes

The color of dried food products is essential to consumers in terms of acceptability and preference^25^. Color changes are related to changes in structural proteins that cause a difference in light-scattering properties on the surface of the marine product, as well as browning reactions^47^. Table 4 shows the mean values of color parameters in squid samples after the drying process at 40 and 60 °C, and both presented a similar trend in the CIE color parameters (L*, a*, and b*). An ANOVA analysis was performed in which the pre-treated samples at 40 and 60 °C were significantly (*p* < 0.05) lighter than the control samples (40 °C → L* = 41.07 and 60 °C → L* = 48.07). The L* value increased for samples pretreated by HPI due to protein changes causing the whitening effect^48,49^. On the other hand, the L* parameter was analyzed concerning the working temperatures (40 and 60 °C), and it was observed that the L* value was lower at a lower temperature in the case of control samples. Increased browning products with the rise in the corresponding drying temperature can explain this decrease in L* on the surface tissue. However, the opposite was true when considering pretreated samples – the ones pretreated and dried at 40 °C showed higher L* than the ones pretreated and dried at 60 °C. In addition, as pressure intensity increased, the L* parameter also increased in most cases (Table 4), which showed statistically significant differences (*p* < 0.05). Similar results were observed in shrimp samples yielding higher values of L* with increasing pressure^50^.

**Table 4:** Effect of processing conditions on color and texture parameters of HPI-dried and control samples.

Run	Color parameters					Texture parameters			
	L*	a*	b*	ΔE	WI	Hardness (gf)	Springiness (mm)	Cohesiveness (–)	Chewiness (mm)
C40	41.07 ± 0.40^h^	12.10 ± 0.02^e^	27.38 ± 0.18^g^	–	33.90±0.28^a^	1977.64 ± 621.98^c^	0.69 ± 0.11^a^	0.58 ± 0.13^a^	909.58 ± 435.85^c^
R1	69.67 ± 0.16^a^	− 3.30 ± 0.03^b^	12.63 ± 0.03^d^	35.67 ± 0.12^a^	67.13±0.15^d^	507.24 ± 199.95^a^	0.90 ± 0.03^c^	0.82 ± 0.01^bc^	419.10 ± 178.27^ab^
R2	74.01 ± 0.09^f^	− 3.84 ± 0.05^a^	10.64 ± 0.03^a^	40.24 ± 0.10^b^	71.66±0.05^c^	723.57 ± 343.79^a^	0.84 ± 0.14^b^	0.77 ± 0.10^b^	560.62 ± 229.79^ab^
R3	70.93 ± 0.13^bc^	− 3.28 ± 0.06^bc^	12.30 ± 0.14^c^	36.82 ± 0.05^a^	68.27±0.07^e^	432.99 ± 156.34^a^	0.86 ± 0.09^bc^	0.82 ± 0.01^bc^	329.72 ± 179.14^a^
R4	71.72 ± 0.23^d^	− 3.25 ± 0.09^bc^	12.07 ± 0.07^c^	37.54 ± 0.18^ab^	69.08±0.18^f^	1050.56 ± 457.74^ab^	0.89 ± 0.03^bc^	0.80 ± 0.04^bc^	630.23 ± 237.33^abc^
R5	76.52 ± 0.05^g^	− 2.77 ± 0.04^d^	14.35 ± 0.22^f^	40.59 ± 0.10^e^	72.34±0.07^b^	420.66 ± 170.52^a^	0.912 ± 0.022^bc^	0.83 ± 0.02^c^	397.21 ± 168.43^a^
R6	71.20 ± 0.08^cd^	− 3.90 ± 0.07^a^	12.61 ± 0.02^d^	37.17 ± 0.07^ab^	68.32±0.07^e^	445.42 ± 250.81^a^	0.90 ± 0.02^c^	0.83 ± 0.02^c^	338.25 ± 183.67^a^
R7	70.47 ± 1.12^b^	− 3.17 ± 0.11^c^	11.43 ± 0.26^b^	36.77 ± 0.88^a^	68.17±0.96^e^	2860.75 ± 189.66^d^	0.86 ± 0.07^bc^	0.79 ± 0.02^bc^	1372.50 ± 626.79^d^
R8	72.66 ± 0.22^e^	− 3.22 ± 0.06^bc^	13.23 ± 0.25^e^	37.85 ± 0.28^b^	69.46±0.09^f^	1566.68 ± 591.25^bc^	0.90 ± 0.01^c^	0.83 ± 0.01^c^	739.92 ± 405.68^bc^
C60	48.07 ± 0.04^F^	11.82 ± 0.04^F^	27.16 ± 0.11^D^	–	40.22±0.02^C^	2577.33 ± 434.55^C^	0.77 ± 0.08^A^	0.70 ± 0.09^A^	2016.24 ± 435.09^C^
R9	58.24 ± 0.38^A^	6.77 ± 0.05^D^	24.27 ± 0.12^A^	11.72 ± 0.31^A^	35.03±0.19^A^	307.80 ± 68.14^A^	0.95 ± 0.01^B^	0.82 ± 0.04^B^	277.14 ± 101.06^A^
R10	57.54 ± 0.24^BC^	6.31 ± 0.15^B^	23.45 ± 0.22^A^	11.57 ± 0.08^B^	41.93±0.42^D^	402.16 ± 178.39^AB^	0.96 ± 0.01^B^	0.82 ± 0.04^B^	320.40 ± 142.14^A^
R11	56.63 ± 0.16^A^	7.84 ± 0.04^E^	24.10 ± 0.26^A^	9.92 ± 0.10^A^	35.76±0.12^A^	534.54 ± 169.12^B^	0.95 ± 0.01^B^	0.82 ± 0.03^B^	422.70 ± 136.68^A^
R12	59.12 ± 0.42^B^	7.59 ± 0.20^E^	25.59 ± 0.24^C^	11.94 ± 0.29^B^	39.02±0.09^E^	484.81 ± 231.68^AB^	0.96 ± 0.03^B^	0.80 ± 0.03^B^	337.51 ± 169.45^A^
R13	59.09 ± 0.38^D^	5.44 ± 0.06^A^	23.30 ± 0.32^B^	13.30 ± 0.23^C^	46.30±0.90^F^	439.85 ± 200.33^AB^	0.96 ± 0.01^B^	0.79 ± 0.04^B^	387.56 ± 187.76^A^
R14	61.37 ± 0.41^C^	6.68 ± 0.11^C^	23.17 ± 0.04^B^	14.80 ± 0.41^B^	41.49±0.23^D^	267.07 ± 130.28^A^	0.94 ± 0.04^B^	0.83 ± 0.03^B^	238.96 ± 88.80^A^
R15	67.68 ± 0.25^E^	4.46 ± 0.02^A^	24.78 ± 0.03^B^	21.02 ± 0.22^D^	48.90±1.11^B^	441.76 ± 281.47^AB^	0.95 ± 0.02^B^	0.80 ± 0.03^B^	439.32 ± 244.45^A^
R16	62.88 ± 0.19^D^	5.82 ± 0.08^A^	24.83 ± 0.11^B^	16.15 ± 0.21^C^	46.91±0.61^F^	414.21 ± 165.50^AB^	0.96 ± 0.012^B^	0.81 ± 0.02^B^	336.28 ± 182.35^A^

Different lowercase letters (a, b, c) show significant differences during the effect of HPI treatment on drying at 40 °C (Run:1–8). Different uppercase letters (A, B, C) show significant differences during the effect of HPI treatments on drying at 60 °C (Run: 9–16). C40: Control 40 °C; C60: Control 60 °C; DT: drying temperature; HP: high pressure; PT: pressure time; OS: osmotic solution.

When considering redness (a* value) and yellowness (b* value), a significant decrease could be observed compared to control samples. These results can be explained by the denaturation of myofibrillar and sarcoplasmic proteins^51^. Similar results were previously reported in the study by Angsupanich and Ledward^52^on cod color, where the myosin was denatured at 100–200 MPa. Also, Cruz-Romero et al^53^. reported similar findings in the case of HP-treated sea bass fillets and HP-treated oysters. Besides, these parameters were analyzed for the working drying temperatures (40 and 60 °C), and it was concluded that the lower the temperature, the lower a* and b* values. Furthermore, when comparing treatments by temperature, statistically significant differences were observed between the experiments for a* and b* values (*p* < 0.05). Statistical analysis of *ΔE* values reflected evident color changes in all pretreated samples compared to control. All the runs in the study exhibited high *ΔE* with values above 9.92 ± 0.10 in the case of runs 9–16 and above 35.67 ± 0.12 for runs 1–8. As previously mentioned by Pérez-Magariño and González-Sanjosé the *ΔE*> 5 indicates that the observed differences were visible to the naked eye^54^. Similarly, Cruz-Romero et al^55^. showed that values of *ΔE* > 12 correspond to very different and strong color differences in the study on oysters. As already discussed, it was demonstrated that the osmotic dehydration pre-treatment under high pressure could inhibit the darkening of the dried samples, as evidenced by the WI (Table 4).

The WI is an important characteristic of many food products, from milk and rice to surimi and pasta^56^. The WI indicates the overall whiteness of food products and may reflect the degree of discoloration during the processing method^57^. HPI pretreatment produces a whitening effect on meat by increasing L* values and decreasing a* values^58^. The WI values for control samples were 33.9 (C40) and 40.2 (C60), respectively (Table 4). Corzo, Bracho, & Marval^59^ reported similar WI = 36.42 values for fresh sardine (*Sardinella aurita*), and Ramirez-Suarez & Morrissey^60^ found WI = 36.42 values for albacore tuna (*Thunnus alalunga*). Applying HPI treatment significantly increased WI values in all samples (67 < R1-R8 < 72 and 35 < R9-R16 < 48) compared to the control samples. The whitening of the flesh was visible to the naked eye (*ΔE*> 5), where HPI-dried samples appeared as cooked. Taskaya et al^61^. investigated how titanium dioxide improves the color of proteins recovered from whole fish. They concluded that the whiteness of restructured fish products based on proteins recovered from entire fish via isoelectric solubilization/precipitation can be similar to that of surimi seafood. Thereby, WI values for processed food products are lacking because natural or processed food products are rarely white enough to fall within the limits of their validity. This way, in the food industry, the frequently used WI is L*, which is often erroneously called “whiteness” instead of “lightness”^56^.

### Texture profile analysis (TPA)

Mean values of textural properties of runs 1 to 16 of dried and then rehydrated (40 °C × 6 h) squid treated by HPI, as well as control samples, are shown in Table 4. The hardness index indicated muscle firmness, cohesiveness was related to viscosity, elasticity, and chewiness, which means tenderness^35^. Significant differences were detected between the control and 1–16 experiments (*p* < 0.05). In this case, the hardness and chewiness parameter values of the control sample were 1977.6 ± 621.9 gf and 909.58 ± 435.8 mm, respectively, being higher than the average of all the treatments. This would indicate a significant decrease of these parameters by 64.3% for hardness and 50% for chewiness on average for all treatments. These results can be due to the damage in the muscular tissue generated by the drying process and the osmotic dehydration, which caused an increase in the formation of pores and cavities (Fig. 2). Consequently, an increase in the ionic force due to the diffusion of NaCl that solubilized the muscle protein could be observed, increasing water retention and tenderness^62,63^. The addition of salts (NaCl) causes displacement in the isoelectric point and consequently changes the pH at which muscles bind water, leading to increased water retention^64^. Therefore, the structure of the rehydrated samples with pre-treatment was softer and less hard than the control samples, except for Run 7, where the high pressure and long pressure time produced a high-salt impregnation; in addition, the drying time at 40 °C was 770 min approx. So, this combination could have caused a crust-thin layer on the sample surface and obtained high-value hardness and chewiness. This showed that the values of firmness expressed as hardness were affected by the pretreatment and the drying temperature. Similarly, when comparing the influence of drying temperature on hardness, the lowest value was reported for Run-5 (420.6 ± 170.5 gf) in the case of 40 °C. In contrast, the highest hardness and chewiness values were noted for the Run-7 (2860.7 ± 1896.6 gf and 1372.5 ± 626.7 mm, respectively). In the case of the temperature of 60 °C the lowest value for hardness and chewiness was obtained in Run-14 (267.0 ± 130.2 gf and 238.9 ± 88.8 mm respectively), and the higher hardness value for Run-11 (534.5 ± 169.1 gf). Moreover, there was no significant difference (*p* > 0.05) between the combinations of pre-treatments (Runs 9–16) on texture parameters. On the other hand, the average values of elasticity (32.8%) and cohesiveness (39.8%) showed a significant increase (*p*< 0.05) compared to the fresh control samples, which shows the HPI-assisted processes affected these values. Similar results were previously reported by Yi et al. in the study on shrimp^65^.

### Rehydration parameters

**Fig. 4:**
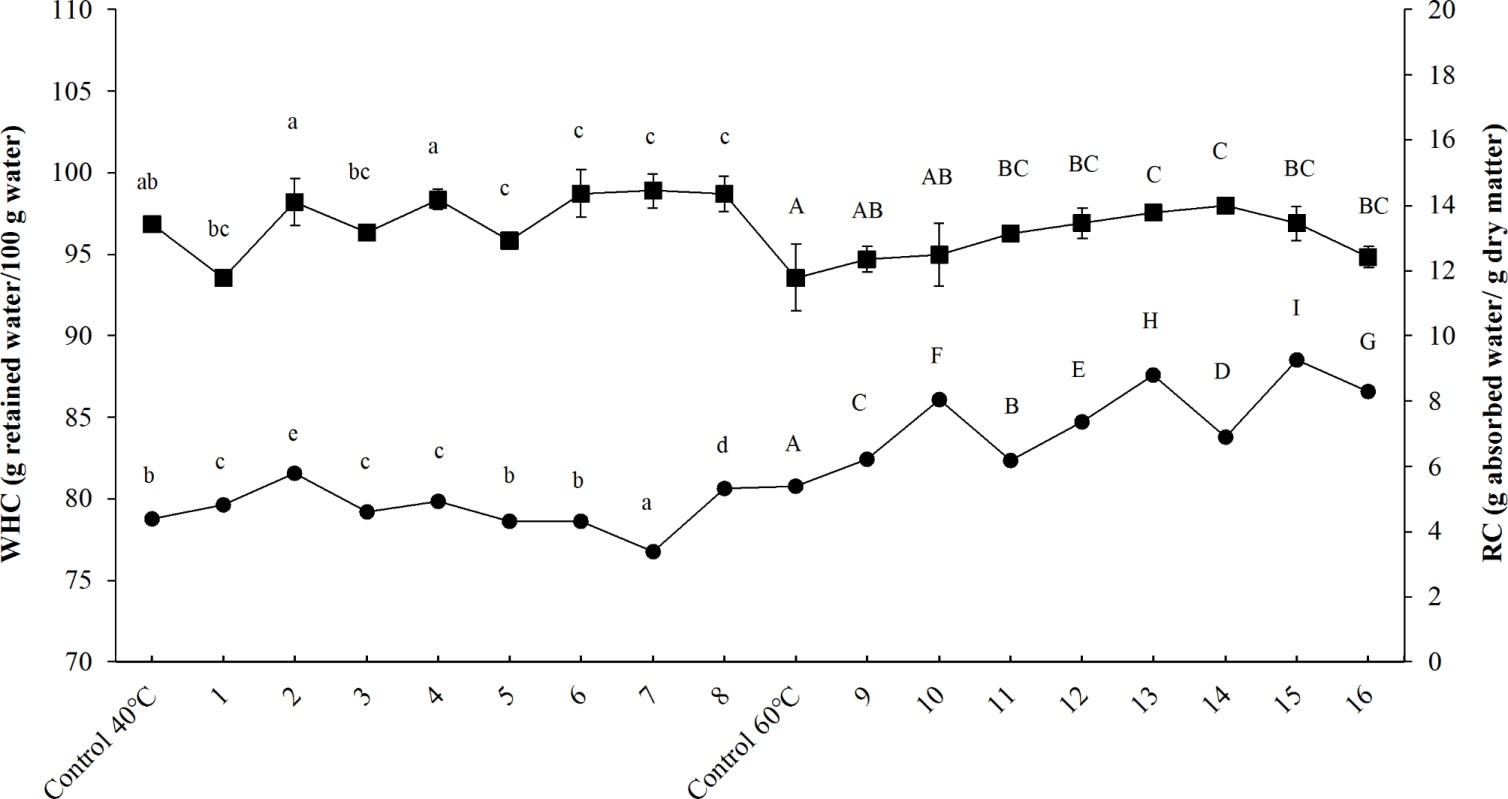
Effect of HPI pretreatment and drying process on water holding capacity (filled square: WHC) and rehydration capacity (filled circle : RC) of jumbo squid slices. Lowercase letters (a, b, c…) show the effect of HPI on drying at 40 °C. Uppercase letters (A, B, C…) show the impact of HPI on drying at 60 °C.

Most dried foods are rehydrated before being consumed. Rehydration can be considered an indirect measure of tissue damage caused by some drying methods^42^. Figure 4 shows the mean values of the rehydration capacity (RC) and water holding capacity (WHC) for the 16 previously treated and control samples. An increase in the drying temperature from 40 (Run 1–8) to 60 °C (Run 9–16) increased the rehydration capacity (RC). The samples dried at higher temperatures were easier to rehydrate, resulting in an increased RC due to more significant structural damage and increased moisture content. On the other hand, when comparing the control sample at 40 °C with the pre-treated samples (Runs 1–8), no clear trend could be observed. However, in the case of Runs 9–16, an increase of RC was reported for pre-treated samples compared to the control. It shows that the drying temperature also had a substantial influence on RC, and changes in RC are related not only to the effect of pre-treatment.

WHC is the ability of structures, e.g., muscles or gels, to resist water loss, and it is essential from the consumer and commercial points of view. It was determined that with an increase in treatment temperature, a simultaneous decrease in WHC values occurred. Values for the 1–8 treatments were, in most cases, significantly higher than those for the 9–16 treatments and the control samples. The slight denaturation of sarcoplasmic proteins could be why the WHC values decrease^66^. The low WHC values can also result from myofibrillar contraction and water transport from the myofilaments to the extracellular space^67^. Protein fragmentation can be listed as one of the main factors responsible for the contraction and/or swelling of myofibrils^68^. WHC parameter of high protein products is significant since many physical properties (e.g., color, texture, and firmness) partially depend on the abovementioned parameter^67^. It was found that drying temperature was the main factor affecting the decrease of the WHC parameter as the temperature increase can aggravate protein denaturation and aggregation, as well as contraction of myofibrils and connective tissue. Moreover, the drying temperature can affect the reduction of extracellular space, intracellular cavities, and channels^7^, which, together with the significant damage to the cellular tissue occurring during high-temperature drying, hindered the water-holding capacity of the samples. Vega-Gálvez et al^69^. applied osmotic dehydration pre-treatment to Jumbo squid drying, and a similar tendency was found for the WHC parameter. On the other hand, when comparing the control samples of both temperatures with the pre-treated samples, it can be observed that the control sample had a lower water-holding capacity value than most of the pre-treated samples. It could be due to the use of NaCl concentrations and high hydrostatic pressure as pre-treatments for drying, which increased the ionic strength since the salt diffusion could solubilize the muscle protein and, in turn, result in a higher water retention capacity due to the changes in the pH at which the muscle bind water^62,63,70^. Therefore, as rehydration capacity increased, water holding capacity decreased due to more significant structural damage at the cellular level, generating, among others, the leaching of soluble solids^71^ and greater positive diffusion of water.

### Changes in microstructure

The Cryo-SEM technique observed microstructural changes in squid samples. Figure 2a and b show the cell structure of 40 °C and 60 °C control samples, respectively, along with Fig. 2c and d, which show the Run-9 and Run-11 samples, respectively.

As for the HPI, the treatment compacted the muscle fibers, causing a rearrangement and a size reduction, so the muscle structure changed significantly. Subsequently, the drying process influenced the tissue structure with various physical changes, such as broken-down cellular membranes and damaged cell walls, appearing in many sites interrupted. So, the cell shapes got more irregular (Fig. 2). Then, a compressed structure was observed. Thus, cell shrinkage was the most important phenomenon, as it caused a significant modification in the sample microstructure and was directly related to water loss during the drying process^25^. The SEM images provided a greater understanding of the processed squid samples by HPI and drying. Deng et al^72^. and Wang et al^73^. also showed changes in dried squid microstructure by different drying methods as well as diverse drying conditions.

## Conclusions

This study revealed that employing high-pressure impregnation as a pretreatment significantly impacted the mass transfer kinetics during drying. The practical implications were evidenced in the shorter drying times and enhanced moisture diffusivity values (4.82–6.59 × 10^−9^ m^2^/s) observed in the HPI-pretreated dried samples. The Logarithmic model presented the best fit among the mathematical models for predicting drying curves. HPI and drying treatment highly influence squid quality, particularly in color changes and increased WI. Regarding the texture profile analysis, the hardness and elasticity decreased on average by 34.2 and 51.9%, respectively, compared to the control sample. Differently, the value of the chewiness increased by 32%, resulting in a product that presented greater tenderness but decreased firmness compared to the unpretreated samples. Also, HPI as a pretreatment did not significantly affect the values of WHC and RC, while the temperature factor did. Based on experimental results and technological and industrial considerations, the run 9 (HPI: 350 MPa, 5 min, 10% NaCl, and drying temperature: 60 °C) can be chosen as the most suitable processing option since this sample demonstrated a salt content appropriate for the average consumer (± 8%) and an ADI equivalent to < 5 g/day of salt according to the WHO. Furthermore, the shortest total processing time − 5 min of HPI and 600 min of drying - may be linked to lower energy consumption, which is highly beneficial for the fishing industry. Additionally, the HPI-dried squid samples (run 9) were easier to rehydrate, leading to an increased rehydration rate due to greater structural damage and enhanced moisture content. However, there was a decrease in water-holding capacity values. Furthermore, the *ΔE* = 11.72 and hardness = 300 gf were the lowest values when compared to the control samples. Finally, the HPI as pretreatment had no greater incidence in the values of WHC and RC, while the temperature factor affected them. Mainly, run 9 (HPI: 350 MPa, 5 min, 10%NaCl, and drying temperature: 60 °C) could be selected as the most acceptable processing variant as this sample showed a salt content suitable for the consumer (± 8%), and the shortest combined processing time (HPI and drying), which could be associated with lower energy consumption. Besides, the microstructure of the HPI-dried squid samples showed compacted cells by SEM. Finally, this study provides information relevant to the broader public regarding the enormous potential in terms of applying high pressure as an impregnation (HPI) technique in seafood ready to be used or for products that are led to further processing like drying, due to the enhanced quality characteristics and reduced drying time.

## Data Availability

Data will be made available by Roberto Lemus-Mondaca upon reasonable request.
